# Blowing Properties and Functionality of Thermoplastic Polyester Film Using Thermally Expandable Microcapsules

**DOI:** 10.3390/polym11101652

**Published:** 2019-10-11

**Authors:** A Ram Pak, Jung Hyun Park, Seung Geol Lee

**Affiliations:** 1 Department of Organic Material Science and Engineering, Pusan National University, Busan 46241, Korea; taylornation@naver.com; 2 Department of Clothing and Textiles, Pusan National University, Busan 46241, Korea; jhpark1391@pusan.ac.kr

**Keywords:** thermally expandable microcapsule, blowing agent, polyester film, mechanical properties, shrinkage, curl, permeability

## Abstract

Blowing film was prepared using a polyester elastomer with thermally expandable microcapsules to investigate its blowing properties and functionality. Film with 11% microcapsule contents showed the lowest specific gravity and the highest blowing efficiency. However, the collapse and merging of blowing cells with 11% microcapsule contents was found by SEM. Therefore, film with 9% microcapsule contents was shown to have better blowing and cell stability than that of film with 11% microcapsule contents. Tensile strength and elongation decreased by increasing microcapsule contents. Film curl and film shrinkage properties were unaffected by microcapsule contents. Water vapor permeability and hydrostatic pressure was decreased by increasing microcapsule contents.

## 1. Introduction

Thermoplastic elastomers (TPEs), which exhibit excellent flexibility and molding processability, are used in various fields such as automobiles, home appliances, and medical applications [1,2,3,4,5]. Among the TPEs, polyester-based TPEs (thermo plastic polyester elastomer, TPEE) are multi-block copolymers in which hard segments of crystalline aromatic polyesters and amorphous soft segments are combined. TPEEs are useful elastomers with excellent mechanical strength, heat resistance, and coating properties [6,7]. Therefore, TPEEs are applied to components and lightweight seats for automobiles, outdoor products where elasticity is important according to the fashion trend of seeking comfort for clothing, and for interior building materials [8]. In addition, TPEEs have been receiving interest by industries as ecofriendly materials because they can save energy and reduce raw material and production costs due to their light weight [9].

The most common method for reducing the weight of TPEEs is by foaming with a blowing agent [10]. The blowing agent is an additive for preparing porous foams such as sponges and styrofoam mixed with a matrix such as a polymer. The blowing agents for foaming are classified into physical blowing agents and chemical blowing agents [11]. Physical blowing agents are additives that form a foaming cell inside a polymer matrix due to the decomposition of carbon dioxide gas or the like dissolved in the polymer matrix at high pressure or the volatilization of a solvent [12]. When a chemical blowing agent (CBA) absorbs heat and reaches a specific decomposition temperature, it decomposes and generates gas, which is used to expand the space inside the polymer matrix to form a foaming cell [13,14,15,16,17]. Thermally expandable microcapsules (TEMs) among chemical blowing agents have been used in various applications since they were developed by Dow Chemical in the 1970s [18,19,20,21]. TEMs are foaming agents in the form of capsules with an average particle diameter of several tens of microns, and are particulate foaming agents with a core-shell structure inside of a low boiling point hydrocarbon and wrapped with a thermoplastic polymer [22]. TEMs have a powder form at room temperature, but when they absorb heat and reach a specific decomposition temperature, the hydrocarbons in the core of the microcapsule expand to form foamed cells [23]. Unlike general CBA, foaming by TEMs does not come out of the surface, so the appearance characteristics during processing are superior to existing foaming processes. In addition, even if a low-viscosity polymer matrix is used, the expansion cell can be maintained through the TEMs so that the collapse of the foam cell is small [24,25,26]. For these reasons, TEMs have been used in many applications to reduce manufacturing cost, reduce weight, create texture, and protect against damage.

The foamed polyester film used in this study is a functional material with moisture permeability and water resistance as a lightweight membrane. In order to study a functional foam film with excellent elasticity and light weight made of elastic polyester material, film specimens were fabricated using elastic polyester material and TEMs for efficient extrusion for the further applications such as outdoor apparel, daily living apparel, and architectural interior materials. Specific gravity, foaming efficiency, tensile strength, elongation, and distribution of foaming cells were analyzed to investigate the foaming properties of the fabricated film. Then, the curl, heat shrinkage, and coefficient of friction of the film required for the post-processing of the film was analyzed. In addition, changes in moisture permeability and water resistance were measured and compared with industrial site standards.

## 2. Materials and Methods

### 2.1. Materials and Specimens Manufacture

KOPEL^®^ (Kolon Plastic Inc., Gimcheon, Korea), which is a copolymerized polymer based on poly(butylene terephthalate-co-tetramethylene ether terephthalate) and Expancel^®^ microspheres (Akzo Nobel Pulp and Performance, Sundsvall, Sweden) was used to prepare a foamed film. Expancel^®^ is available with expansion temperatures in the range of 80–190 °C. Pellet type of TEMs was used to improve the dispersion in this study. A Thermo Scientific Process 11 (Thermo Fisher Scientific, Karlsruhe, Germany), a parallel co-rotating extruder with a length-to-diameter ratio of 5, was used to prepare polyester-based thermoplastic elastomer foam films. The manufacturing process conditions of the extruder were a speed of 50 RPM at 210 °C. 

### 2.2. Characterizations

#### 2.2.1. Specific Gravity and Blowing Efficiency

A densitometer (MD-300s, Alfa Mirage, Osaka, Japan) with standard test methods of ASTM D792 was used to measure the specific gravity and foaming efficiency of the foamed polyester film. The specific gravity of each sample was measured five times at room temperature and the average value was obtained. Foaming efficiency was determined by Equation (1) using the specific gravity of the polyester polymer before foaming and the specific gravity measured after foaming.

(1) $$\mathrm{Foaming}\;\mathrm{efficiency}\;\left(\%\right)=\frac{SG_{0}}{SG_{f}}\times 100$$

where *SG*_0_ is the specific gravity of the polyester polymer before foaming and *SG_f_* is the specific gravity of the polyester polymer after foaming, respectively.

#### 2.2.2. Tensile Strength and Elongation

The tensile strength and elongation of the foamed polyester film were measured by a universal test machine (DWU2100A, Dongwon SM, Busan, Korea) with extensometer. The standard test methods of ASTM D882 was used to measure the tensile strength and elongation of the foamed polyester film. The sample dimensions were set to 2.54 cm (width) × 5.08 cm (height). The strain rate was 50 mm/min. Tensile strength and elongation were measured five times and the average value was obtained.

#### 2.2.3. Surface Analysis

Surface images were taken using a scanning electron microscope (S-4700, Hitachi, Tokyo, Japan) to analyze the size and distribution of the foamed cells of the foamed polyester film.

#### 2.2.4. Linear Dimensional Change 

Standard test methods of ASTM D1204 were used to determine the dimensional changes with the temperature required for the application of polyester elastic films to indoor and outdoor products. The heat shrinkage rate was measured at a temperature of 40 °C and 60 °C after 12 h and 24 h, respectively. The size of test specimens was 25 cm × 25 cm. Conventional electric oven (LDO-150F, Daihan Labtech Co. Ltd., Namyangju-si, Korea) was used to test the dimensional change. The linear dimensional change was calculated by Equation (2).

(2) $$\mathrm{Linear}\;\mathrm{change}\;\left(\%\right)=\frac{D_{f}-D_{0}}{D_{0}}\times 100$$

where *D_f_* is the final length of specimen after test and *D*_0_ is the original length of specimen, respectively. 

#### 2.2.5. Curl

Curling was measured to identify curl occurrences that could greatly reduce the processability of the polyester elastic film. The measuring method was to place the film on a flat bottom, use a cutter knife to draw an X mark on the film with a length of 10 cm, and then measure the degree of bending of the part of the cut film on the cutter blade with a ruler or Vernier caliper. 

#### 2.2.6. Friction Coefficient

The friction coefficient was measured with standard test methods of ASTM D 1894 to verify the friction and wear resistance of polyester elastic film. A horizontal glass plate was used to obtain the dynamic friction coefficient. The size of test specimens was 12 cm × 12 cm. The friction coefficients were determined by a friction coefficient tester (FCMS170, Neoplus Co. Ltd., Seoul, Korea).

#### 2.2.7. Water Vapor Permeability and Water Resistance 

Moisture permeability was measured with standard test methods of KS K 0594 using the calcium chloride method. The diameter of test specimens was 7 cm. The determination of resistance to water penetration was tested with standard test methods of KS K ISO811 and KS K 0592 by hydrostatic pressure method. All tests were carried out by KATRI, Busan, Korea.

## 3. Results and Discussion

### 3.1. Specific Gravity and Blowing Efficiency

**Figure 1:**
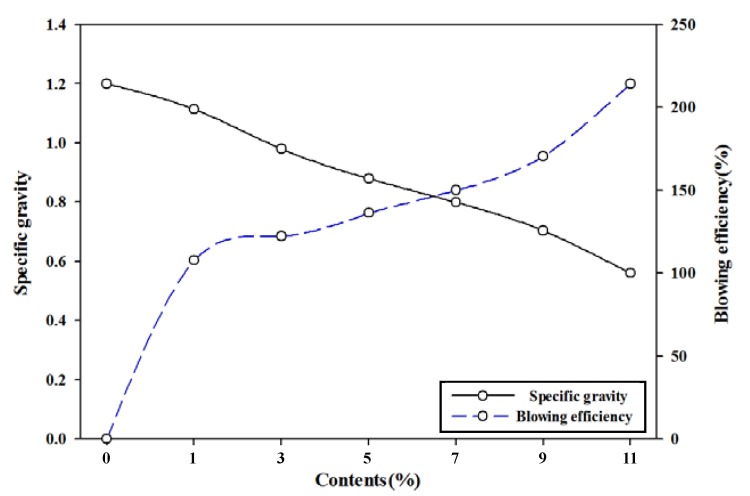
Specific gravity and blowing efficiency of polyester thermoplastic film with various contents of thermally expandable microcapsules.

As shown in Figure 1, specific gravity decreased continuously as the TEMs content of the foamed polyester film increased. In particular, the decrease in specific gravity was sharp when the TEMs content was 7% or more compared with polyester film prepared without using a blowing agent. Foaming efficiency increased with the increasing TEMs content of the foamed polyester film. As a result, the lowest specific gravity and the highest foaming efficiency of the foamed polyester film were achieved with TEMs content of 11%. 

### 3.2. Foam Cell Shape and Distribution

**Figure 2:**
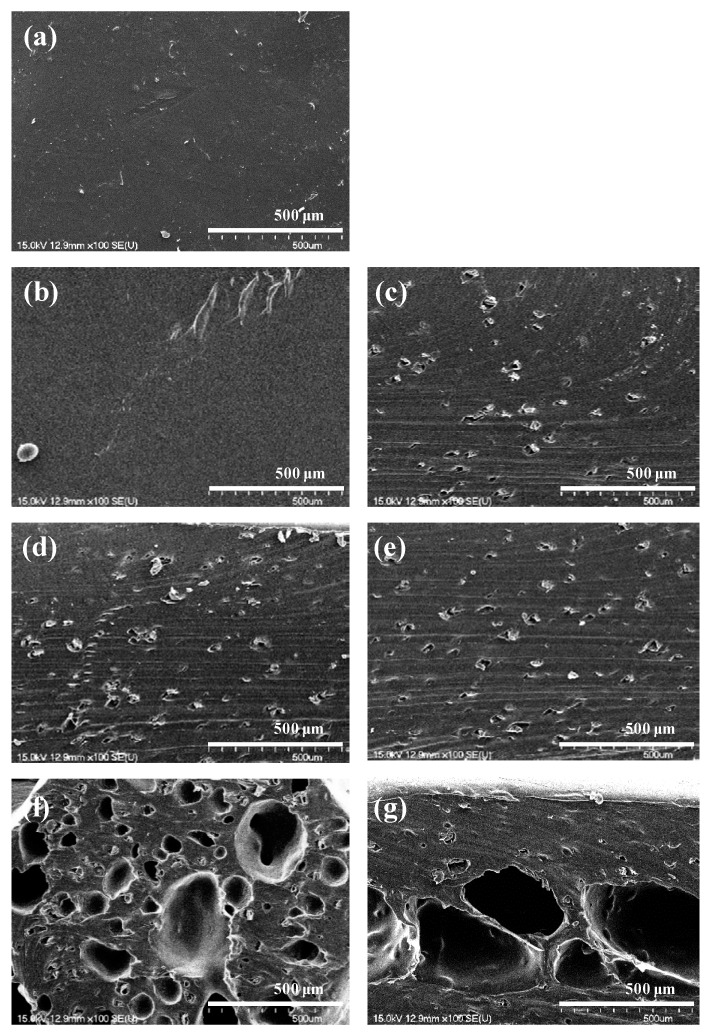
SEM images (X100) of polyester thermoplastic film with various contents of thermally expandable microcapsules: (**a**) 0%, (**b**) 1%, (**c**) 3%, (**d**) 5%, (**e**) 7%, (**f**) 9%, and (**g**) 11%, respectively.

**Figure 3:**
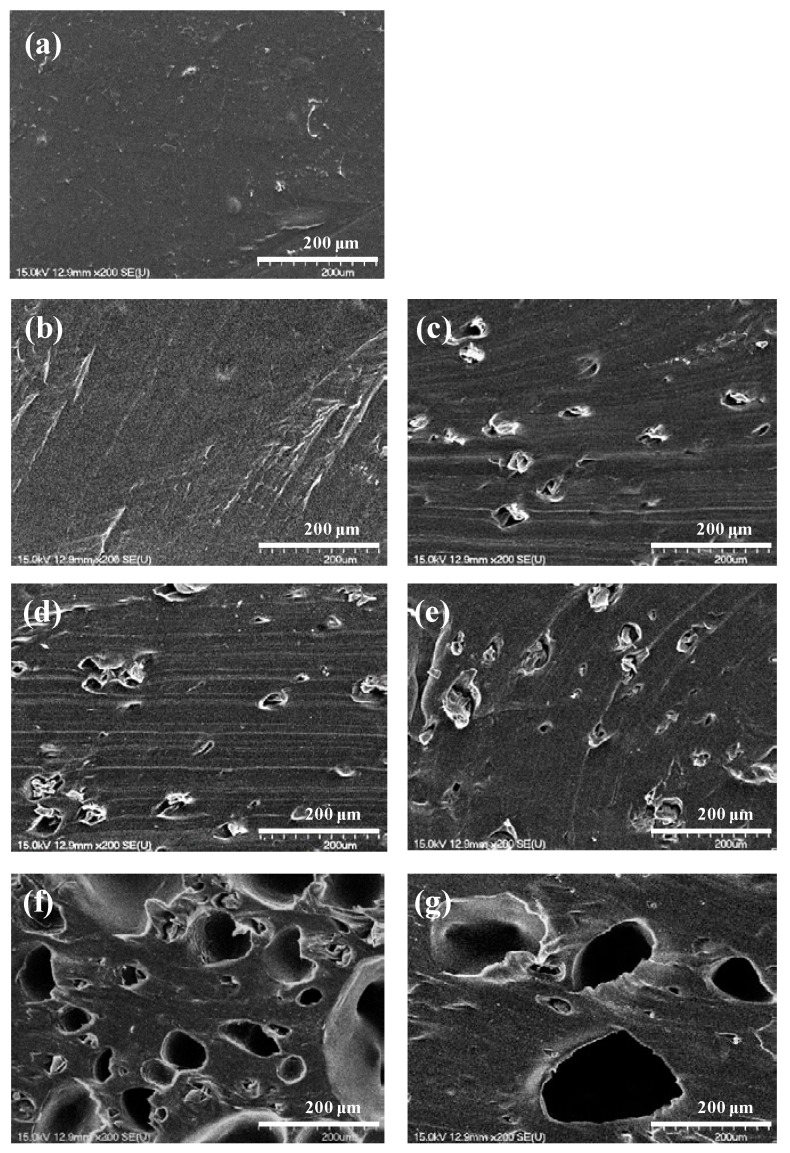
SEM images (X200) of polyester thermoplastic film with various contents of thermally expandable microcapsules: (**a**) 0%, (**b**) 1%, (**c**) 3%, (**d**) 5%, (**e**) 7%, (**f**) 9%, and (**g**) 11%, respectively.

The surface was analyzed by SEM to confirm the foam cell shape and distribution of the foamed polyester film according to the TEMs content. As shown in Figure 2 and Figure 3, no foaming cells were observed with the addition of 1% TEMs and for films without foaming agents. In case of 1% TEMs content, we assumed that most TEMs were blown in the extruder before it reached the nozzle exit. The formation of foam cells in the polyester film was observed starting from 3% TEMs content. The number of foam cells increased from 5% TEMs content, and the growth of foam cells was observed with 7% TEMs content. The highest expansion cell growth was observed with TEMs content of 9% or more. However, foam cells at 9% TEMs content showed uniform foaming and growth, but foam cells at 11% TEMs content were collapsed and fused. Thus, it was found that the foaming stability was not good for the polyester film with 11% TEMs content. Therefore, in view of the shape of the foam cells, the foaming stability of foam cells in the polyester film is highest with TEMs content of 9%.

### 3.3. Tensile Strength and Elongation 

**Figure 4:**
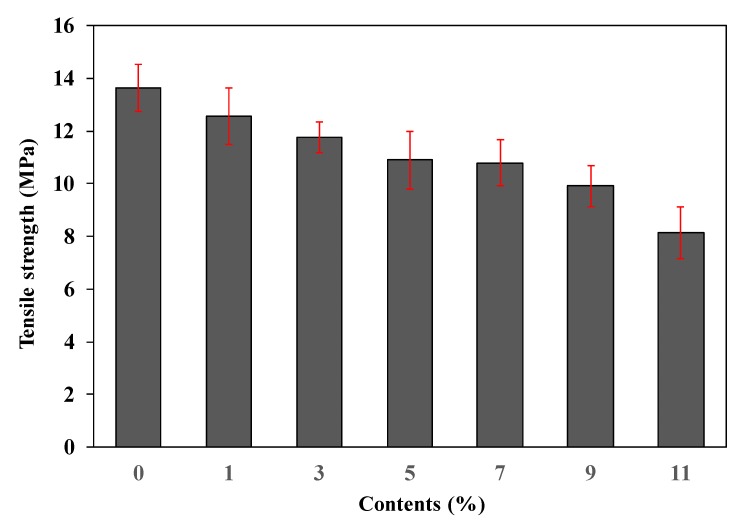
Tensile strength of polyester thermoplastic film with various contents of thermally expandable microcapsules.

The tensile strength of the foamed polyester film with various contents of TEMs was measured. As shown in Figure 4, the tensile strength decreased as the TEMs content increased. However, the tensile strength of the foamed polyester film with TEMs content of 11% was 8.14 ± 0.98 MPa, which is less than the minimum required tensile strength of ~9.8 MPa for film applications [27].

**Figure 5:**
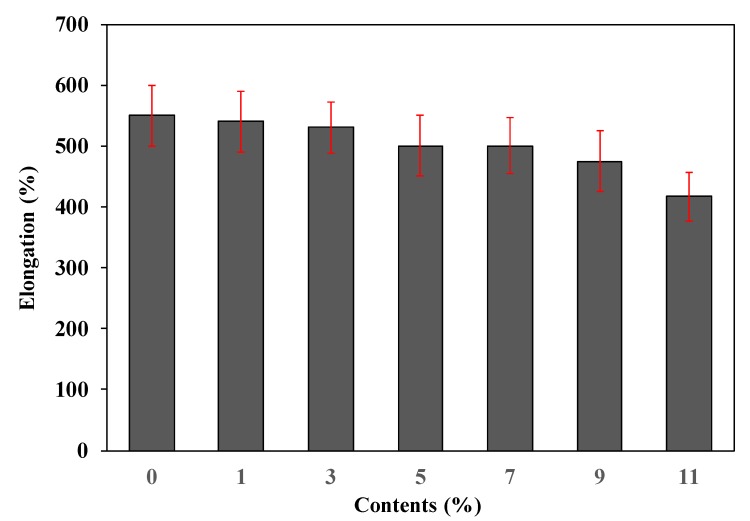
Elongation of polyester thermoplastic film with various contents of thermally expandable microcapsules.

As shown in Figure 5, the elongation of the foamed polyester film decreased as the TEMs content increased. The decrease in elongation with increasing TEMs content increased when the TEMs content was 11%. This is because film with 11% TEMs has low foaming cell uniformity [23].

### 3.4. Linear Dimensional Change 

In order to use the film produced for its purpose, it is important to have dimensional stability in various temperature environments because polyester film can be applied to various industries such as clothing, construction, and automobiles. Therefore, linear dimensional change in conditions of 40 °C and 60 °C was measured after 12 h and 24 h, respectively. According to the results of linear dimensional change of polyester thermoplastic film with various contents of TEMs, the foamed polyester film did not undergo heat shrinkage even with high TEMs content. These results show that the foamed cells are not reduced in size or collapse with a given temperature. Thus, the foamed polyester film is considered to have excellent dimensional stability regardless of the increase in TEMs content. Otherwise, the thermal properties of polymer may affect to the dimensional change of the specimen [28,29,30]. 

### 3.5. Curl

**Figure 6:**
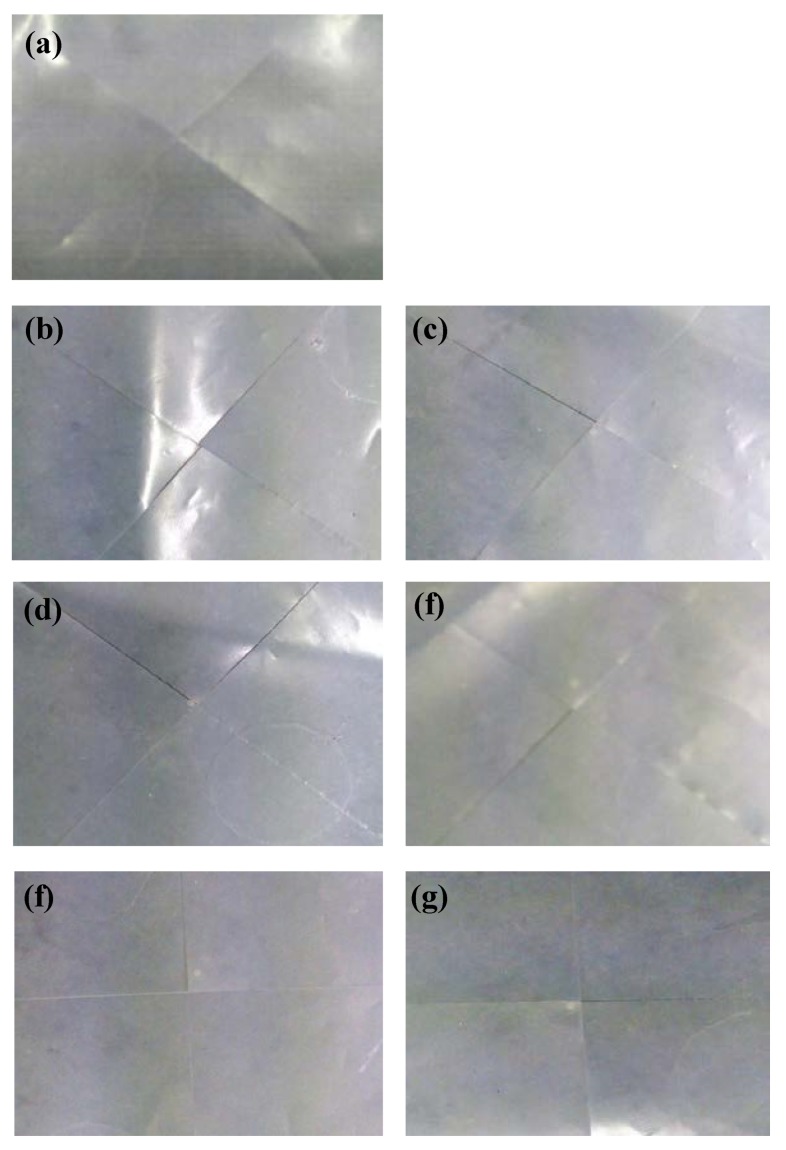
Polyester thermoplastic films with various contents of thermally expandable microcapsules after curl test.

Curl refers to a phenomenon in which the surface of the film floats while forming a bend at the bottom when material such as a film or sheet is cut out and spread on the floor. When the degree of curl increases, processing efficiency due to the folding phenomenon is reduced. In order to prevent curling, alternatives such as the selection of processing temperatures, use of various additives, and reverse winding are used in the production process. In particular, the elastic material has a disadvantage in that it may be more vulnerable to curl due to the elastic properties of the material during post-processing, so it is necessary to identify and solve these problems. Therefore, the curl of the foamed polyester was measured and the results are shown in Figure 6. According to the results of curl of polyester thermoplastic film with various contents of TEMs, as the TEMs content increases, no curling phenomenon occurs. 

### 3.6. Coefficient of Kinetic Friction 

**Figure 7:**
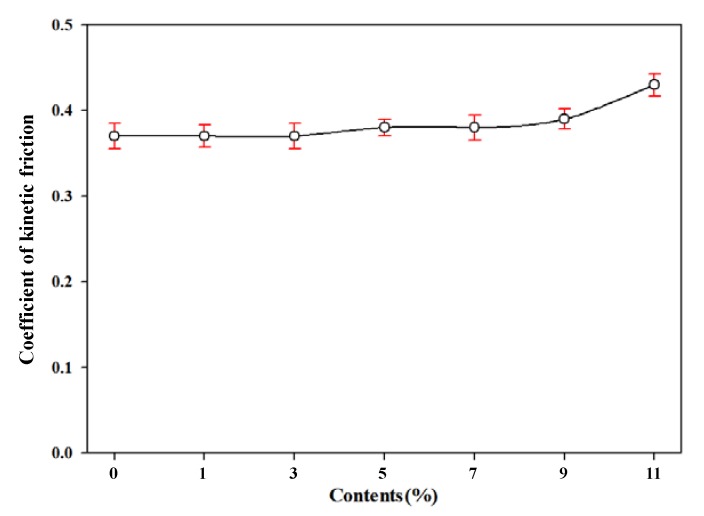
Coefficient of kinetic friction of polyester thermoplastic film with various contents of thermally expandable microcapsules.

The higher the coefficient of friction is indicated the greater the degree of friction of the material. In particular, if the coefficient of friction is high, the processability and durability of the film can be decreased. The formation of foam cells could increase the friction of foamed polyester film using TEMs, so the coefficient of kinetic friction was measured in this study. As shown in Figure 7, the coefficient of kinetic friction of the foamed polyester film increased as the amount of TEMs increased, especially at 11% TEMS content. We assume that relatively larger forming cells growth can caused irregular and rough surface at 11% TEMs content. It will cause relatively large changing of the kinetic friction coefficient of foamed film at 11% TEMs content. However, since the difference in the kinetic friction coefficient value according to the TEMs content was small, it was determined that the amount of TEMs content does not have a significant effect on the degree of friction and durability of the film. Since the required kinetic friction coefficient of polyester film in the film industry is 0.35 to 0.40 [31], foamed polyester film with up to 9% TEMs content is applicable to film applications.

### 3.7. Water Vapor Permeability and Water Resistance

**Figure 8:**
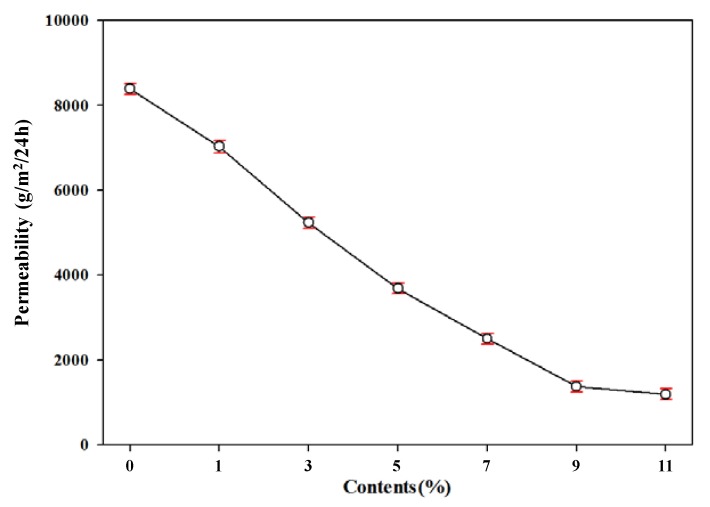
Water vapor permeability of polyester thermoplastic film with various contents of thermally expandable microcapsules.

The moisture-permeable waterproof function is an important property to prevent water from the outside and to discharge moisture inside to the outside [32,33]. As shown in Figure 8, moisture permeability rapidly decreased as the TEMs content increased. We assumed that permeability functionality of polyester film is decreased with increasing TEMs contents by loss of the molecular structure of the thermoplastic polyester film by foaming. For outdoor applications, moisture permeability should be over 5000 g/m^2^/24 h. If moisture permeability is 2500 g/m^2^/24 h or more, it can be applied to daily clothing. It can be applied to interior materials with moisture permeability of 700 g/m^2^/24 h [34]. Therefore, foamed polyester film with up to 3% TEMs content can used in outdoor applications, with up to 7% TEMs content can be applied to daily living apparels, and with more than 9% TEMs content can be applied to architectural interior materials.

**Figure 9:**
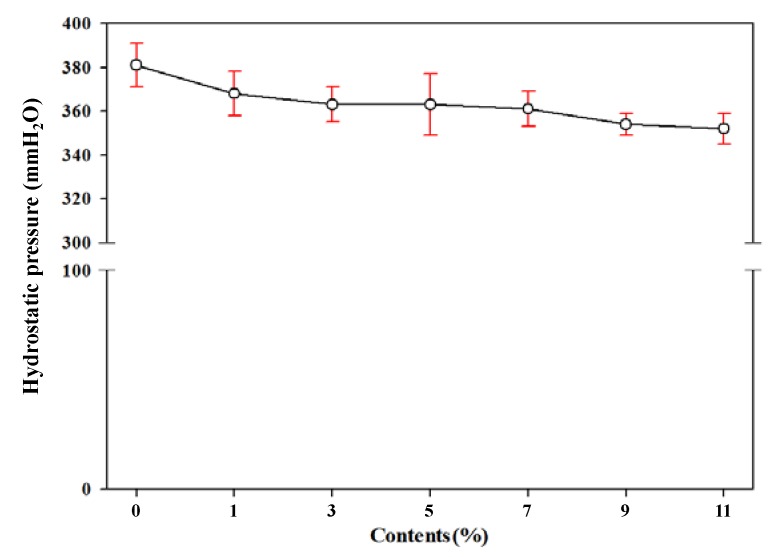
Hydrostatic pressure by low pressure tester of polyester thermoplastic film with various contents of thermally expandable microcapsules.

In addition, a hydrostatic pressure water test was performed to measure the waterproof performance. The hydrostatic pressure method used to test waterproof or leakage of fiber, synthetic leather, wrapped paper, textiles, films, etc. The low-pressure water tester applied the pressure of 10 ± 0.5 cmH_2_O/min to the sample to measure the water resistance of the specimen. As shown in Figure 9, with a low hydrostatic pressure water tester, the hydrostatic pressure decreased with increasing TEMs content, but the difference was not significant. If the hydrostatic pressure is over 300 mmH_2_O, it can be applied as a target material for low hydrostatic pressure conditions [34]. Therefore, there is a slight decrease in the low hydrostatic pressure performance by increasing the TEMs content, but the results satisfied the standard under all conditions. 

**Figure 10:**
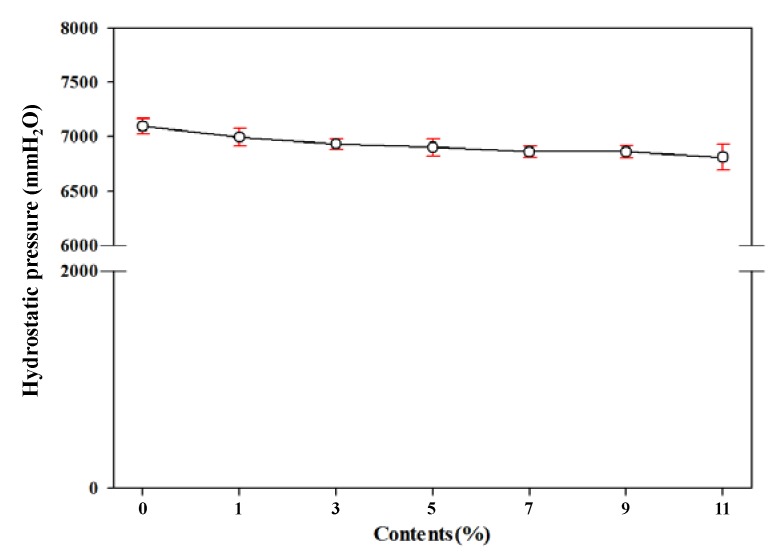
Hydrostatic pressure by high pressure tester of polyester thermoplastic film with various contents of thermally expandable microcapsules.

To investigate the waterproof performance in more severe conditions, we conducted a high hydrostatic pressure water test of the foamed polyester film. The high-pressure tester applied the pressure of 85 ± 5 mL/min to the sample to measure the water resistance. As shown in Figure 10, the hydrostatic pressure decreased with increasing TEMs content, but no significant decrease was observed. If the hydrostatic pressure ranges from 5000 to 30,000 mmH_2_O, it can be applied as a target material for high hydrostatic pressure conditions [34]. Thus, the results in all conditions satisfied the industrial requirements well. 

## 4. Conclusions

In this study, lightweight foamed polyester film was prepared using TEMs as foaming agents to study foaming characteristics and conduct functional tests. As the content of TEMs increased, specific gravity decreased and foaming efficiency increased. Tensile strength and elongation decreased with the formation of foam cells inside the polymer with increasing TEMs content. As a result of observing the distribution and morphology of the foam cell with SEM analysis, the specific gravity of the foamed polyester film was lowest with 11% TEMs content, but it was confirmed that foam stability was reduced due to foam cell collapse or cell fusion due to excess foaming agent. Linear dimensional change, curling, and friction coefficients were measured for the post-processing aspects of the foamed polyester film. Heat shrinkage and curling of the foamed polyester film with increasing TEMs content was not observed. The difference in the friction coefficient according to the TEMs content was also not significant. Water vapor permeability decreased by increasing TEMs content. Hydrostatic pressure decreased slightly by increasing microcapsule contents. Lightweight polyester film was achieved by foaming using TEMs with reasonable mechanical properties, water vapor permeability and water resistance for the various applications in functional textile fields.
